# Kombucha: An Old Tradition into a New Concept of a Beneficial, Health-Promoting Beverage

**DOI:** 10.3390/foods14091547

**Published:** 2025-04-28

**Authors:** Dhuelly Kelly Almeida Andrade, Boying Wang, Emília Maria França Lima, Sergei Konstantinovich Shebeko, Alexey Mikhailovich Ermakov, Valentina Nikolaevna Khramova, Iskra Vitanova Ivanova, Ramon da Silva Rocha, Manuela Vaz-Velho, Anthony Nhamo Mutukumira, Svetoslav Dimitrov Todorov

**Affiliations:** 1 ProBacLab, Laboratório de Microbiologia de Alimentos, Departamento de Alimentos e Nutrição Experimental, Food Research Center, Faculdade de Ciências Farmacêuticas, Universidade de São Paulo, São Paulo 05508-000, SP, Brazil; dhuelly@gmail.com (D.K.A.A.); emiliamflima@usp.br (E.M.F.L.); ramonrocha@usp.br (R.d.S.R.); 2 School of Food and Advanced Technology, Massey University, Auckland Campus, Auckland 0745, New Zealand; nikiwang1979@hotmail.com (B.W.); tony.mutukumira@gmail.com (A.N.M.); 3 Faculty of Bioengineering and Veterinary Medicine, Don State Technical University, 1 Gagarina Sq., Rostov-on-Don 344000, Russia; shebeko_sk@mail.ru (S.K.S.); amermakov@ya.ru (A.M.E.); 4 Department of Food Production Technology, Volgograd State Technical University, V.I. Lenin Avenue 28, Volgograd 400005, Russia; hramova_vn@mail.ru; 5 Department of General and Applied Microbiology, Faculty of Biology, Sofia University St. Kliment Ohridski, 8 Dragan Tzankov Blvd., 1164 Sofia, Bulgaria; iskrai3@yahoo.com; 6 CISAS—Center for Research and Development in Agrifood Systems and Sustainability, Instituto Politécnico de Viana do Castelo, 4900-347 Viana do Castelo, Portugal; mvazvelho@estg.ipvc.pt; 7 Department of General Hygiene, I.M., Sechenov Forst Moscow State Medical University, Moscow 119991, Russia

**Keywords:** kombucha, tea kombucha, health benefits, safety

## Abstract

Kombucha is an ancient, fermented beverage that has gained increasing popularity worldwide due to its potential health benefits. Its origins trace back to China, from where it spread across Asia and Europe before reaching the modern global market. The fermentation of kombucha is mediated by a Symbiotic Culture of Bacteria and Yeasts (SCOBY), comprising yeasts, acetic acid bacteria, and lactic acid bacteria. The microbial consortium plays a crucial role in the production of organic acids and bioactive metabolites, shaping the sensory characteristics of the beverage. Given the growing interest in kombucha as a functional beverage, this study aims to explore its historical background, fermentation process, and microbiological composition, including key yeasts, acid acetic bacteria, and lactic acid bacteria and their interactions. Additionally, we describe the potential health effects of kombucha, particularly its antimicrobial and antioxidant activity, the probiotic potential of the strains associated with kombucha, and safety considerations while also addressing the risks associated with its consumption. Although several studies suggested that kombucha may have antioxidants, antimicrobial, and probiotic properties, as well as contribute to gut microbiota regulation and immune system support, there is significant variability in the composition of the beverage, especially in artisanal preparations. This variability poses challenges in standardizing its potential effects and ensuring consistent safety. The risk of contamination further underscores the importance of adhering to strict sanitary production standards. To scientifically validate its health benefits and guarantee safe consumption, further research with larger sample sizes and robust methodologies is essential. The findings of this study will contribute to a deeper understanding of the functional properties of kombucha and provide scientific support for its safe and beneficial applications.

## 1. Introduction

### History of Kombucha

Kombucha is increasingly becoming popular due to the perceived health and benefits of fermented beverages. The origins of kombucha can be traced to around 220 B.C. in northeastern China during the Qin Dynasty [1]. According to Chinese folkloric sources, kombucha was called “Tea of Immortality”. The beverage was prized for its perceived health benefits [2] due to inherent antioxidants, antimicrobial compounds, and immunostimulants. The health benefits are derived from infused tea or other substrates, the consequences of the tea-based substrate, and the metabolic roles of bacteria and yeast cultures [3]. The origins of kombucha are somewhat unclear, but it is widely accepted that kombucha originated from China to other parts of Asia and later to Europe through trade routes such as the Silk Road between China and Europe [4].

Until the end of the 19th century, kombucha was not well known outside of Asia. In the early 20th century, kombucha spread to Russia, where it became known as “tea kvass”. Kombucha was spread over Europe and the rest of the world and gained a variety of names: “Manchurian Mushroom Tea”, “Tea Fungus”, “Kargasok Tea”, “Grib tea kvass”, “Indian Tea Fungus”, “Manchu Fungus”, “Teakwass”, “Tea Beer”, and many others [5]. It gained popularity, particularly in rural areas, and was often a homemade product. In the following years, kombucha consumption spread to Eastern Europe and Germany, where it gained popularity due to its perceived health benefits, safety, and appealing sensory characteristics [6].

Kombucha was introduced to the United States in the late 20th century due to the popularity of its health benefits. Interestingly, kombucha somehow gained popularity in the USA during the HIV/AIDS epidemic in the 1980s and early 1990s, as it was perceived to boost the immune system and increase T-cell counts [7]. However, its popularity decreased after a 1995 report by the Centers for Disease Control and Prevention (CDC) that linked kombucha to two cases of severe metabolic acidosis, one of which was fatal [8].

**Figure 1:**
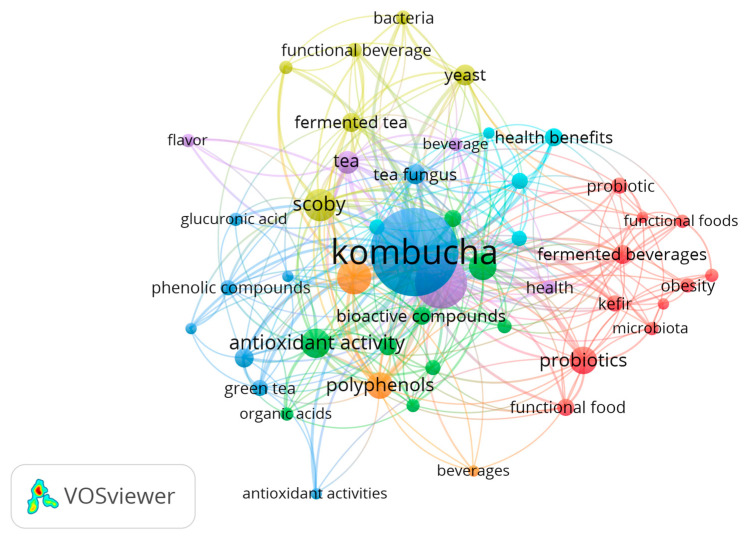
Bibliometric analysis related to co-occurrence of terms “kombucha” and “health” from 2000 to 2025, elaborated with Scopus metadata using VOSviewer 1.6.20 Software.

During the COVID-19 pandemic, kombucha was in the spotlight as it was perceived to boost the immune system, and the beverage is characterized by potential beneficial antimicrobial and antiviral properties [9]. In the early 21st century, kombucha experienced a resurgence in popularity, driven by growing interest in functional products, especially those containing probiotics and fermented foods. Moreover, commercial kombucha was portrayed as a healthy drink, and commercial production increased significantly. Literature bibliometric analysis linked health properties and kombucha (Figure 1). Kombucha became widely available in health food outlets, supermarkets, and even mainstream grocery stores [10].

Today, kombucha is a global product. It is brewed both commercially and at home, with various flavors and formulations. The global kombucha market was valued at approximately $5 billion in 2025 and it continues to grow [11]. Despite its popularity, it is important to note that many health claims associated with kombucha lack robust scientific evidence, and the beverage should be therefore consumed in moderation.

## 2. Microbiology

### 2.1. Fermentation Process and Metabolic Pathways

**Figure 2:**
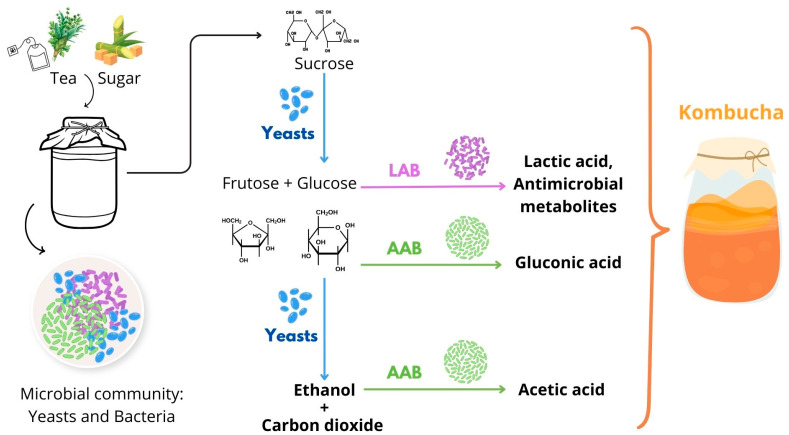
Metabolic pathways involved in kombucha fermentation. The schematic illustrates the microbial interactions between yeasts, acetic acid bacteria (AAB), and lactic acid bacteria (LAB).

Kombucha is made by fermenting sweetened tea with a Symbiotic Culture of Bacteria and Yeast (SCOBY). From a microbiological standpoint, the primary components of kombucha include acetic acid bacteria (AAB), yeast, organic acids, and other metabolites [6]. Small amounts of lactic acid bacteria (LAB) have been reported in Kombucha, and their role has not been fully studied [6]. Kombucha can be described as a fermented beverage in which the fermentation involves the conversion of sugars (added to the tea infusion) into ethanol by yeasts and acetic acid by the AAB. Yeasts, part of the SCOBY consortium, metabolize sucrose into glucose and fructose, which are then metabolized into ethanol. AAB convert ethanol into acetic acid, which decreases pH, thereby giving kombucha its characteristic sour taste [6,12]. In addition, yeasts produce carbon dioxide (CO_2_), which imparts fizzy characteristics to the beverage (Figure 2).

Kombucha fermentation is a two-step process. The primary fermentation process starts with the preparation of the sweetened tea, traditionally prepared using infused black, green, or oolong tea and unrefined sugar. The SCOBY is added to the infused sweetened tea. The yeast in SCOBY begins to metabolize the monosaccharide sugars, producing ethanol and carbon dioxide. At the same time, AAB in SCOBY converts the ethanol into acetic acid, which decreases the pH and gives kombucha its characteristic tangy flavor [13]. In the secondary fermentation, which is optional, the formation of specific flavors can be obtained. Additional ingredients such as fruits, herbs, or spices may be then added. When kombucha is bottled and sealed, the remaining yeast continues to ferment any residual sugars, resulting in carbonation and further reduction in pH [14]. Kombucha has been prepared for centuries in various geographical regions with different primary substrates, sweetened with sugars, as well as other natural sweeteners as carbohydrate bases, which may explain microbiological variations in kombucha. Understanding the microbial interactions in kombucha can help improve its quality and consistency [6].

### 2.2. Main Microorganisms Present in Kombucha

The microbial diversity in kombucha is broad, involving various species of bacteria, predominantly AAB and yeast, but the involvement of LAB has been reported.

#### 2.2.1. Yeast

Yeast is one of the primary microorganisms involved in the fermentation of kombucha. The yeast ferments sugars into ethanol and carbon dioxide, which further contributes to the fizziness of the beverage [15]. Yeast species vary between kombucha beverages due to several factors, including environmental factors and even contamination between starter cultures [6]. The genera *Zygosaccharomyces* and *Dekkera* are frequently found in kombucha [12,14]. However, in the metagenomic analysis performed by Kaashyap et al. [16] in kombucha, the most dominant yeast genera were *Starmerella*, followed by *Galiella*, *Hanseniaspora*, *Zygosaccharomyces*, and *Microidium*. These results demonstrate microbial diversity enriched with organic acid-producing microorganisms in the fermented beverages.

In addition, yeast representatives from the genus *Zygosaccharomyces* have been frequently reported as part of the SCOBY consortium. *Zygosaccharomyces* spp. play a crucial role in the fermentation process due to their high tolerance to acidic environments, which explains their presence in kombucha, where the pH of the final products can range from 2.5 to 3.5 [12,17]. *Zygosaccharomyces bisporus* is tolerant to high sugar and acidic environments, making it well-suited for kombucha fermentation [18] (Table 1).

Due to its ability to produce acetic acid and ethanol, *Brettanomyces bruxellensis* is important for the fermentation process and the development of the complex flavor profile of the beverage. Species such as *Candida stellata* and *Candida krusei* have also been reported in kombucha. They contribute to the fermentation process and the production of various flavor compounds [18].

#### 2.2.2. Acid Acetic Bacteria (AAB)

Bacterial species are predominantly represented by AAB, including species from the genera *Acetobacter*, which produces acetic acid, and *Gluconobacter*, which produces gluconic acid [14,16]. In a metagenomic analysis performed by Kaashyap et al. [16] in kombucha, the most dominant genera of AAB was *Acetobacter*, followed by *Komagataeibacter*, *Gluconobacter*, *Swingsia*, and *Asaia*. The role of AAB is associated with the conversion of ethanol into acetic acid, which reduces the pH, contributes to specific aromas, and acts as a preservative. *Komagataeibacter rhaeticus*, an AAB species, is one of the most prevalent in kombucha and is known for its ability to produce acetic acid and bacterial cellulose, which forms the characteristic biofilm/cellulosic pellicle or SCOBY [18]. In addition, *Acetobacter aceti*, another common AAB, plays a crucial role in converting ethanol produced by yeast into acetic acid, contributing to the acidic taste of kombucha [16]. *Gluconobacter oxydans* is involved in the oxidation of glucose to gluconic acid, impacting the acidity and flavor profile of kombucha [18] (Table 1).

#### 2.2.3. Lactic Acid Bacteria (LAB)

The presence of minor levels of lactic acid has been reported in kombucha, but their role is not well understood. Some of the most reported LAB in kombucha are representatives from the former genus *Lactobacillus*, which since 2020 was reclassified into several new genera [19]. Furthermore, all former lactobacilli were presented with their new systematic names [19] and abbreviated according to recommendations from Todorov et al. [20]. By redefining over 250 species into 25 distinct genera, it was targeted and achieved greater precision in understanding the taxonomy, physiology, and metabolic traits of these bacteria [19]. From the perspective of academic point of view, this enables more systematic studies on probiotics, fermentation, and gut microbiota, fostering advancements in health and microbiology where relations between physiology, biochemistry, and genetics can be related to health performance and other benefits. For industry, the refined classification improves the selection of specific bacterial strains for use in food production, pharmaceuticals, and agriculture, enhancing product efficacy and innovation. Overall, this reclassification bridges scientific insight with practical applications for broader societal impact.

In kombucha, lactobacilli are associated with the production of lactic acid, which contributes to the tangy flavor, and some of them may have potential health-promoting benefits [21,22,23]. The presence of different groups of LAB has been reported in artisan preparations [12], but there are limited studies on their role in the fermentation of kombucha (Table 1).

LAB are generally considered safe microorganisms where they have been accorded the Generally Recognized As Safe (GRAS) status. However, this popular perception may be misleading and clearly needs to be investigated, as several LAB can be associated with food spoilage microorganisms. Moreover, physiologically, LAB produce D- and L- forms of lactic acid, where D-lactic acid has been associated with acidosis in babies and small children [24]. Thus, good sanitation standards need to be followed when preparing kombucha, particularly at an artisanal level. Although less frequently reported, species such as *Lactiplantibacillus plantarum* and *Lacticaseibacillus casei* have been reported in kombucha as well. The interactions between the microorganisms are complex and dynamic and can involve different symbiotic relations between yeasts, AAB, and LAB as part of the SCOBY. LAB are involved in the modulation of pH and production of flavors and varieties of antimicrobials, including lactic acid, diacetyl, low molecular metabolites, bacteriocins, carbon dioxide, and hydrogen peroxide [25]. These antimicrobial metabolites, in combination with phenolic compounds as part of the tea infusion, play an essential role in the beneficial properties of kombucha [26], an advantage that will be discussed later in this paper (Table 1).

### 2.3. Microbial Interactions in Kombucha

Microbial co-metabolism is one of the principal ways of interaction and even synergistic coexistence. With the focus on cooperative metabolic processes where bacteria and yeasts share resources and contribute to the environment’s balance, kombucha is an example of mutual benefits between different organisms and highlights how metabolic byproducts influence the growth and activity of other microorganisms. Moreover, the structural development of SCOBY is a complex process where biofilm generation is crosslinked with cellulose production, a process that involves a microbial diver community. In addition, cell-to-cell signaling interactions and *quorum sensing* are essential in the mentioned processes, emphasizing how chemical signals regulate collective behaviors such as biofilm formation, metabolic adjustments, and the role of signal molecules in synchronizing community responses.

Factors such as temperature, oxygen availability, and substrate concentration significantly influence microbial activity and (fermentation) dynamics of the fermentation process and formation of the final products [27]. All that, in combination with the quality of the tea infusion, the appropriate quantity of added carbohydrates, and the composition of SCOBY, are essential to produce kombucha with desirable characteristics. Understanding microbial interactions and fermentation dynamics is essential for optimizing kombucha production, ensuring consistent quality and safety of the final beverage, and enhancing its health benefits.

Interactions of microbial activities of yeast, AAB, and LAB are involved in the formation of SCOBY. Symbiosis, formation of microbial communities, biofilm formation, and *quorum sensing* are only a few interactions between a variety of microbial species involved in the formation of SCOBY and the fermentation of kombucha. The interactions between these microorganisms are essential for the successful fermentation of kombucha.

One of the main interactions in the fermentation of kombucha is symbiosis, where yeasts produce ethanol and carbon dioxide, which are then utilized by AAB to produce acetic acid. The symbiotic relationship helps maintain the balance of the microbial community and the overall quality of kombucha [18] and ensures balanced and efficient fermentation. Yeasts, particularly *Saccharomyces cerevisiae*, produce the enzyme invertase, which hydrolyzes sucrose into glucose and fructose. These monosaccharides are then utilized mainly by yeasts and even further by LAB. At the same time, yeasts ferment glucose and fructose to produce ethanol and carbon dioxide, while AAB actively oxidizes ethanol to acetic acid [28]. The final processes not only contribute to the specific acidic taste of kombucha but also contribute to maintaining the microbial balance by preventing the overgrowth of yeasts associated with a reduction in pH. Interaction between microbial components involved in the fermentation of kombucha results in the formation of SCOBY, where AAB are actively involved in the formation of cellulosic biofilms, which are essential for their survival, including the yeast and LAB in the kombucha matrix. Cellulosic biofilms provide a protective environment and facilitate efficient nutrient exchange and microbial communication. The presence of yeasts can enhance biofilm formation by providing not only ethanol but also other metabolites, including vitamins and peptides that can be utilized by AAB and LAB [6,14].

The interaction between yeasts and AAB can lead to metabolic shifts. For example, the presence of AAB can increase the invertase activity and fermentative metabolism of *Saccharomyces cerevisiae*, leading to higher production of ethanol and other metabolites [28]. This metabolic interplay ensures a continuous supply of substrates for both yeasts and AAB, promoting a stable fermentation process.

Biofilm formation is the principal process associated with the formation of SCOBY. The production of bacterial cellulose by the representatives of genus *Komagataeibacter* in kombucha is essential for the formation of cellulosic biofilm that further can support the growth of all microbial components of the SCOBY, creating a stable environment for the fermentation process [29].

*Quorum sensing* is a crucial process in the fermentation of kombucha, which involves communication between microbial cells through signaling molecules, allowing them to coordinate their activities based on the cell density of all microorganisms involved in the formation of SCOBY during fermentation [30]. Therefore, *quorum sensing* plays a fundamental role in regulating these interactions. For instance, AAB uses *quorum sensing* to regulate biofilm formation, which further controls their survival and function in the kombucha matrix. The biofilm provides a protective environment and facilitates efficient nutrient exchange and microbial communication [31]. Due to *quorum sensing*, SCOBY represents a balanced microbial community, ensuring that the metabolic activities of the bacteria and yeast are synchronized, leading to a consistent and high-quality fermentation process.

Some of the microbial metabolites produced by the kombucha microbial community involved in the regulation of *quorum sensing* processes are Acyl-homoserine lactones (AHLs), Autoinducer-2 (AI-2), and Farnesol. AHLs are commonly produced metabolites by Gram-negative bacteria; however, they can also be expressed by some AAB. AHLs assist *quorum sensing* processes, permitting bacterial constituents of SCOBY to coordinate activities, including biofilm formation and acetic acid production [32]. AI-2 is a signaling molecule involved in regulation processes by Gram-positive and Gram-negative bacteria. It was suggested that in kombucha, AI-2 assists in interspecies communication, ensuring that the various microbial communities, including bacteria and yeast, work together harmoniously. Farnesol is produced by yeast, which acts as a *quorum-sensing* mediator associated with the inhibition of the growth of certain bacterial species. This facilitates maintaining the balance inside the microbial community of kombucha by preventing any single microbial species from overgrowth and dominating the fermentation process [33]. The microbial diversity of kombucha is a key factor in its unique characteristics for the fermentation process and is essential for the formation of sensory characteristics and further providing combinations of metabolites with potential health-promoting benefits. The presence of various AAB, LAB, and yeasts contributes to the fermentation process, flavor development, and the perceived beneficial properties of the beverage.

## 3. Health Benefits

Kombucha has over a century-long history, and Eastern traditional medicine suggests beneficial regarding immune boosting and improvement after infectious diseases [9] as it is perceived to offer several health benefits. Several beneficial properties of kombucha involve yeast, acetic acid, LAB, and their metabolites, in addition to a variety of phenolic compounds obtained from tea infusion. However, more research is needed to confirm the effects of the compounds, including appropriate animal models and clinical trials. Some studies suggest that kombucha may have antioxidant properties, support gut health, and provide antimicrobial effects due to the presence of organic acids and polyphenols [6]. Additionally, the probiotics present in some kombuchas can contribute to a healthy gut microbiome [21,22].

Probiotic properties are often associated with kombucha; however, the implications need to be investigated. Beneficial probiotic properties are frequently mentioned in different fermented products. Probiotic properties are characteristics of specific microorganisms that can be part of fermented food products, including kombucha. However, fermented food products themselves are not probiotics. Naturally fermented products have variable microbial composition since these are not controlled fermentation processes. To be considered probiotic, specific microorganisms need to be taxonomically identified, possess proven specific beneficial properties, and the benefits confirmed in appropriate animal and even controlled human clinical trials as well as the presence of appropriate levels of viable cell numbers [34,35]. Therefore, kombucha microbiota needs to be analyzed to determine their probiotic properties by systematic scientific studies. This hypothesis was investigated by Bogdan et al. [23], who characterized five strains of LAB isolated from kombucha using morphological and molecular techniques. The results indicated a resemblance to *Pediococcus pentosaceus* strains, which produce a bacteriocin and are tolerant to bile salt, exhibiting significant probiotic potential.

In general, based on available evidence, kombucha is a fermented beverage with potential beneficial properties. The fermented beverage contains various beneficial strains, including AAB, LAB, and yeast, and some of these could be good probiotic candidates. The beneficial strains (potential probiotics) can be associated with supporting gut health by balancing the gut microbiome, improving digestion, and potentially alleviating gastrointestinal issues such as Irritable Bowel Syndrome (IBS) and inflammatory bowel diseases [36,37].

Kombucha, especially when made with green tea, is rich in antioxidants. Green tea is well-studied regarding its beneficial properties, with a focus on different phenolic compounds related to their antioxidant properties [38]. Phenolic compounds in green tea, particularly catechins like epigallocatechin gallate (EGCG), offer numerous health benefits. These compounds are potent antioxidants that help neutralize harmful free radicals in the body, thereby reducing oxidative stress and lowering the risk of chronic diseases [39]. Studies have shown that green tea phenolics can improve cardiovascular health by reducing blood pressure, cholesterol levels, and the risk of heart disease [40]. Additionally, they exhibit anti-cancer properties by inhibiting the growth of cancer cells and inducing apoptosis [41]. Green tea phenolics also have anti-inflammatory effects, which can help manage conditions like arthritis and inflammatory bowel disease [42]. Further, they support metabolic health by enhancing fat oxidation and improving insulin sensitivity, making them beneficial for weight management and diabetes prevention [43].

Antioxidants contribute to the neutralization of free radicals, reducing oxidative stress, and, consequently, can potentially lower the risk of chronic diseases such as heart disease and cancer [44]. Some studies of animal models suggested that kombucha can protect against liver toxicity [45]. Studies have shown that the antioxidant activity of green tea is significantly higher than that of black tea due to its higher catechin content [46]. Additionally, green tea antioxidants have been reported to improve skin health by protecting against UV radiation and reducing signs of aging [47].

The above-described features of the composition of biologically active substances of kombucha and its basic pharmacological effects also suggest a beneficial impact on the kidneys and urinary system [48]. Thus, the nephroprotective effect of kombucha was demonstrated in rats with diabetic nephropathy induced by streptozotocin. In the study, kombucha significantly improved the course of nephropathy, which was confirmed by a decrease in signs of kidney damage according to the biochemical and histological assessments [49]. Also, the antioxidant and protective effect of kombucha on the kidneys of rats fed on a hypercholesterolemic diet [50]. In another study [51], kombucha reduced trichloroethylene-induced kidney damage in rats. Results of rats given kombucha showed a decrease in lipid peroxidation and oxidative stress in kidneys, as well as normalization of the blood residual nitrogen level. The results indicated that kombucha may be effective in patients with kidney diseases, as well as preventing and protecting kidneys from intoxication and nephrotoxicity.

The bacteria of SCOBY (AAB and LAB) are related to the production of acetic acid and lactic acid during kombucha fermentation, which have antimicrobial properties. Organic acids can inhibit the growth of spoilage and food-borne pathogens at low pH levels, including *Escherichia coli* and *Salmonella* spp. [52]. For instance, the growth of *E. coli* and *Salmonella* spp. is significantly reduced at pH below 4.0 [53]. In another study, Kaewkod et al. [54] reported the antimicrobial activity of kombucha against some enteropathogenic bacteria, including *Vibrio cholerae*, *Shigella dysenteriae*, *E. coli*, and *Salmonella typhimurium*. Antibiotic activity against the pathogens was attributed to the presence of organic acids, such as acetic acid and benzoic acid. The bactericidal effects of acidic environments are due to the disruption of cellular processes and denaturation of proteins [55]. Additionally, the presence of organic acids, such as those found in fermented food products, can enhance the bactericidal effects under low pH conditions.

This makes kombucha a potentially safe beverage that can help maintain a healthy microbial balance in addition to improving the food safety of the final fermented beverage. These findings suggest that kombucha could be a valuable natural resource in combating enteropathogenecity, with the potential to contribute to gastrointestinal health [37]. The catechins found in green tea-based kombucha, such as epigallocatechin-3-gallate (EGCG), can boost metabolic rates and enhance fat oxidation [56]. Bioactive compounds, such as polyphenols and acetic acid in kombucha, have been linked to the alleviation of chronic inflammation and other health conditions, including heart disease, diabetes, and arthritis [9].

## 4. Safety

While kombucha is generally considered microbiologically safe for most people, it should be consumed in moderation. Kombucha prepared in artisanal conditions may contain variations in microbiological composition, and some opportunistic pathogens or spoilage microorganisms may survive. Excessive consumption of the beverages can lead to adverse effects such as acidosis or digestive issues. Kombucha is generally safe when prepared under good sanitary conditions; therefore, it is important to ensure that kombucha is prepared as such to avoid contamination [10].

However, there is a risk of contamination with pathogenic microorganisms if hygiene is not maintained during fermentation. Cases of contamination with *Aspergillus* and *Penicillium* species have been reported [57]. Some of the molds can be associated with the production of mycotoxins, which may present health hazards [58]. Home-brewed kombucha poses a higher risk of contamination compared to commercially produced kombucha due to poorly controlled environments [59].

The microbial composition of kombucha can enrich the final product with acetic acid, as metabolic products of activity of AAB. Excess production of acetic acid can be associated with metabolic acidosis. Few rare reports have focused on metabolic acidosis associated with excessive acetic acid kombucha consumption. This condition occurs when the body produces excess acid or when the kidneys are not removing enough acid from the body [60].

The presence of LAB is associated with the production of lactic acid. The safety concern can be caused by the production and formulation of D-lactic acid, produced by some species of LAB. Some examples include *Lactobacillus delbrueckii*, a well-documented bacterium due to its ability to produce high levels of D-lactic acid [61]. *Lpb. plantarum*, *Limosilactobacillus reuteri*, or *Lactobacillus acidophilus* can also produce significant amounts of D-lactic acid, especially under certain fermentation conditions [62]. These LAB species are important in various fermentation processes, and their ability to produce D-lactic acid can be a health challenge in food fermentation processes and related to acidosis, especially for young children [24,61,63]. A few case studies have documented severe acidosis in individuals consuming large quantities of kombucha, highlighting the need for moderation [63,64].

Most of the fermented food products are considered low in allergenicity and only in isolated cases can allergic reactions occur. Moreover, ingredients of tea used as a base for the preparation of kombucha may be associated with some allergic reactions. Thus, some individuals may experience allergic reactions to kombucha, possibly due to the presence of yeast or other microbial components or components of the primary ingredients. Moreover, the preparation of kombucha and added sugar can be contradictory for individuals with sugar intolerance. In some instances, symptoms associated with the consumption of kombucha can include itching, swelling, and respiratory issues. It is important for individuals with known allergies to yeast or mold to exercise caution [65].

Toxicity, in some cases, may be associated directly with the primary material or microorganisms involved in the fermentation processes. Using ceramic or lead-glazed containers for brewing kombucha can lead to leaching lead into the beverage, leading to a risk of food poisoning. Thus, appropriate hygienic conditions need to be used in the fermentation process of kombucha and using glass, stainless steel, or food-grade plastic containers to avoid this risk are recommended [66].

Poor hygiene practices may result in the contamination of fermented beverages by some molds due to acidic conditions. Mycotoxin-producing molds are a potential concern in kombucha fermentation as they may be associated with a specific metabolic ability to produce toxins that can contaminate the beverage. By nature, mycotoxins are secondary metabolites produced by certain molds, such as *Aspergillus*, *Penicillium*, and *Fusarium* species [67], and they can be associated with the spoilage of kombucha. The molds can grow on the surface of the kombucha culture (SCOBY) or in the fermentation vessel if proper hygiene and fermentation conditions are not maintained. Normally, these types of mycotoxin-producing molds are not commonly found in kombucha SCOBY. However, in some instances, contamination with mycotoxin-producing molds can occur if the SCOBY or the brewing environment is exposed to mold spores, a consequence of poor hygiene production conditions. The toxins can pose health risks, including immunosuppression, carcinogenicity, and other toxic effects [58].

To minimize the risk of mycotoxin contamination, it is essential to maintain a clean brewing environment, use high-quality ingredients, and regularly inspect SCOBY for any signs of mold growth [66]. Ensuring proper fermentation conditions, such as maintaining the correct pH and temperature, can also help prevent the growth of mycotoxin-producing molds [68].

Prevention of mycotoxin-poisoning when consuming low-quality kombucha is an important area for scientific research. For this purpose, it is desirable to use laboratory models of the gastrointestinal tract (GIT), which allow for assessing the bioavailability of mycotoxins and their interaction with intestinal epithelium, microbiota, and probiotics [69]. These models can also be used to select optimal binders for inhibiting the effects of mycotoxins. Thus, the effectiveness of chitosan and three cellulose derivatives [70], as well as three biosorbents [71], in binding some of the most well-known mycotoxins were studied under model GIT conditions. It was also shown in GIT models that microbial enzyme degradation of mycotoxins is a preferred method than the use of absorbents [72]. This method of mycotoxin degradation is characterized by high specificity, environmental friendliness, and improved safety characteristics [73,74]. It is possible to obtain genetically modified probiotic strains, especially those containing recombinant enzymes that ensure the degradation of mycotoxins [75]. Including such strains in the composition of the kombucha, a starter can eliminate the problem of mycotoxin contamination when preparation is compromised.

Kombucha is produced through a fermentation process involving yeast, and as such, ethanol has evolved. Kombucha naturally contains small amounts of ethanol. The beverage can contain alcohol ranging from 0.5% to 1.5%, which is generally described as low alcohol. Alcohol content in home-brewed beverages can be higher [76]. Commercially produced kombucha is regulated to ensure alcohol content remains below 0.5% to be classified as a non-alcoholic beverage [77]. However, considering that alcohol can be present in the final products needs to be carefully considered when the beverage is offered to children or individuals with specific nutritional or religious restrictions [78].

## 5. Health Benefits Versus Risks

Principal risks for consuming kombucha can be associated when the beverage being produced on an artisanal scale, where no appropriate control of the fermentation processes is performed. Home-made products are often accepted as healthier and safer; however, this can be misleading since a lack of food processing knowledge or experience and inappropriate production conditions can lead to contamination and compromised quality and safety of the prepared food products, including kombucha. To prevent contamination, guidance for producers and consumers on the safe preparation, handling and storage of kombucha was described by Murphy et al. [66]. Thus, industrial production of kombucha involves strict sanitary protocols, use of approved primary materials and ingredients, control of fermentation and post-fermentation processes, distribution, and commercialization, subject to additional control from the appropriate government food control institutions. In essence, it is imperative that kombucha should be produced under approved Good Manufacturing Practices (GMPs) and Hazard Analysis Critical Control Points (HACCPs).

Labeling products with health-promoting beneficial properties related to improving gut health without supporting data is misleading to consumers and against international norms on health claims. Some kombucha products may contain beneficial microorganisms that may support gut health by promoting a balanced microbiota, but this must be supported by scientific data. However, individuals with compromised immune systems should consult healthcare providers before consuming fermented food products with viable microbial cultures and even clinically proven pharmaceutical probiotic preparations.

Antioxidant properties of kombucha are related to the specific high phenolic compounds related to the primary material used for production, either green or black tea. The numerous beneficial properties of tea and inherent phenolic compounds are well documented and were part of the tradition and modern health (pharmaceutical and medical) practices [9]. The antioxidant compounds can contribute to the reduction of oxidative stress and help with some inflammatory conditions. Kombucha may be a beneficial beverage when consumed responsibly and prepared under proper sanitary conditions. However, despite these benefits, excessive consumption of kombucha can have the opposite effects, and thus, overconsumption should be avoided to prevent potential adverse effects [79]. Thus, it is known that kombucha can be useful for the kidneys due to the presence of flavonoids and other polyphenolic compounds in tea [80]. liu [81,82]. Thus, moderation and balanced consumption are key terms concerning any beneficial fermented food product recommended by traditional or modern health practices to be applied aimed at health promotion.

Kombucha is not a clinical drug but a traditional fermented beverage with a long history of containing potential health-promoting compounds. Awareness of potential risks, such as microbial contamination, acidosis, allergic reactions, and lead poisoning, is crucial for safe consumption. Consumption in moderation and adherence to safe brewing practices are key to enjoying the health benefits of kombucha without adverse effects.

## 6. Clinical Studies

Traditional medicine recommends the consumption of kombucha and has proven potential benefits from the consumption of fermented beverages. Moreover, in the last few decades, clinical studies have investigated the potential health benefits of kombucha [35,83].

Diabetes in different forms is a real silent pandemic of the 21st century. Western medicine suggests different combinations between pharmaceutical control and appropriate nutritional restrictions. However, appropriate nutri-pharmaceutical approaches can be an interesting approach for improving the status of hyperglycemic disorders. Application of balanced nutrition, food products with therapeutic benefits, including specific fermented foods, have always been part of traditional medicine, and even Hippocrates postulated that food needs to be balanced to improve the health status of patients [84]. Anti-hyperglycemic effects are suggested as one of the health-promoting effects of kombucha [85].

A pilot clinical study conducted by researchers at Georgetown University, the University of Nebraska-Lincoln, and MedStar Health (USA) explored the effects of kombucha on blood sugar levels in people with type 2 diabetes. In the reported study, 12 participants who consumed either kombucha or a placebo for four weeks, followed by an eight-week washout period before switching to the alternate beverage, observed benefits. The results showed that kombucha significantly lowered average fasting blood glucose levels from 164 mg/dL to 116 mg/dL after four weeks, whereas the placebo did not produce a significant change [85,86].

Another study investigated the effects of a four-week kombucha supplementation in healthy individuals, dividing participants into a kombucha group (n = 16) and a control group (n = 8). Researchers collected stool and blood samples to analyze the human microbiome and inflammation markers. By the end of the experiment, they observed an increase in fasting insulin and HOMA-IR in the kombucha group, while the control group exhibited a reduction in HDL cholesterol levels. The kombucha intervention had modest effects on gut microbiome composition and biochemical parameters. These findings highlight the need for studies with larger sample sizes and longer durations to better understand the impact of kombucha consumption on gut microbiota modulation and its implications for human health and disease outcomes [87].

## 7. Other Benefits

Kombucha, particularly when made with green tea, is rich in different antioxidants. Antioxidants help neutralize free radicals, reducing oxidative stress and potentially lowering the risk of chronic diseases. Although most evidence comes from in vitro and animal studies, these findings suggest that kombucha may offer antioxidant benefits to humans as well [85]. While these studies provide promising insights into the potential health benefits of kombucha, it is important to note that more extensive and rigorous clinical trials are needed to confirm these properties. However, as mentioned before, the main weakness in these studies is to establish a clear link between variations in the microbial composition of kombucha and health benefits.

Some of the components in the green tea infusion have health promoting benefits, including weight loss [88]. A randomized controlled trial examined the impact of green tea kombucha not only on weight loss but also showing beneficial properties regarding alleviating inflammation and salivary microbiota in individuals with excess body weight [89]. The study involved two groups: one group followed a caloric restriction diet and consumed 200 mL of green tea kombucha daily, while the control group followed only the caloric restriction diet. Both groups showed significant reductions in weight, body mass index, and body fat. However, the kombucha group also demonstrated a significant reduction in lipid accumulation products and lower levels of inflammatory markers such as IL-6 compared to the control group [90].

Moreover, a systematic review evaluated the effects of kombucha consumption on obesity-related comorbidities and gut microbiota. The review included 15 studies and found evidence suggesting that kombucha consumption can be beneficial for controlling obesity and associated comorbidities. The studies highlighted the potential of kombucha to modulate gut microbiota, which plays a crucial role in weight management and metabolic health [91]. The previously mentioned benefits were studied in animal models, and it was suggested that kombucha, particularly green tea kombucha, may help prevent obesity by reducing gut dysbiosis, supporting intestinal barrier function, and reducing adipose tissue inflammation. These effects are thought to be mediated by the bioactive compounds in kombucha, such as polyphenols and organic acids [90]. While these studies provide promising insights into the potential benefits of kombucha for weight management, it is important to note that more extensive and rigorous clinical trials are needed to confirm these effects in humans. As with any dietary supplement, kombucha should be consumed in moderation, and individuals with specific health conditions should consult their healthcare providers before incorporating the fermented beverage into their diet.

Some of the beneficial microorganisms from kombucha can aid in digestion by enhancing the balance of gut bacteria. A healthy gut microbiome is essential for efficient digestion and nutrient absorption. Moreover, these beneficial microorganisms can help in the processes of breakdown of food, non-digestible components, production of vitamins, and support the gut lining [92]. The diverse microbial community in kombucha can contribute to a more balanced gut microbiota and contribute to improved digestion, enhanced immune function, and reduced risk of chronic diseases.

Chronic inflammation in the gut can lead to various gastrointestinal disorders, such as Irritable Bowel Syndrome (IBS) and Inflammatory Bowel Disease (IBD). Beneficial microorganisms, some of them part of the kombucha, may assist in the processes of reduction in inflammation by modulating the immune response and maintaining the integrity of the gut barrier [56]. By improving gut microbiota balance, which plays a crucial role in immune function, kombucha can contribute to supporting the immune system, modulate the immune response, reduce inflammation, and enhance the resistance to infections [9]. Moreover, since the gut is home to a significant portion of immune cells of the body located in the gut-associated lymphoid tissue, beneficial microorganisms from kombucha can interact with gut-associated lymphoid tissue and consequently promote the production of antibodies and enhance the immune response [93].

Moreover, some of the bacterial cultures can produce different antimicrobials, including diacetyl, low molecular antimicrobials, CO_2_, H_2_O_2_, bacteriocins, and other antimicrobial proteins [94] that can contribute not only to the safety of the fermented beverage but also conferring potential benefits to consumers [52]. Acetic acid is one of the primary organic acids in kombucha, produced by AAB during fermentation. The organic acid contributes to sour taste and has antimicrobial properties [95]. Lactic acid produced by LAB contributes to the tangy flavor and acts as a natural preservative [96]. Acid is produced by the oxidation of glucose by AAB, which has been shown to have antioxidant properties [36]. Glucuronic acid is reported to aid the detoxification processes in the liver by binding to toxins and facilitating their excretion [45,93].

Kombucha, especially when made with green tea, is rich in antioxidants such as polyphenols. Antioxidants help neutralize free radicals, reducing oxidative stress and supporting overall immune health. Studies have shown that antioxidants can enhance immune response by protecting immune cells from damage [65]. While direct clinical studies on the effects of kombucha on immune function are limited, there is some evidence from related research showing that kombucha consumption improved immune responses and increased the activity of macrophages, which are crucial for fighting infections. Human clinical trials on antioxidants for immune health are well-documented [97,98].

Some of the polyphenols and antioxidants recorded in kombucha include epigallocatechin gallate, flavonoids, and catechins, which are powerful antioxidants [41]. During fermentation, their levels can be modified, enhancing the antioxidant capacity of kombucha. Epigallocatechin gallate is a type of catechin with strong antioxidant properties, particularly abundant in green tea-based kombucha [46]. Flavonoids are compounds that contribute to the antioxidant activity of kombucha and are derived from the tea leaves used in the fermentation process [44,99]. Moreover, kombucha can contain various B vitamins, including B1 (thiamine), B2 (riboflavin), B6 (pyridoxine), and B12 (cobalamin), which are produced by the yeast and bacteria cultures during fermentation [100].

Other bioactive compounds in kombucha include ethanol, amino acids, and specific enzymes. Small amounts of ethanol are produced by yeast during fermentation [15]. While typically low, ethanol contributes to the antimicrobial properties of kombucha. Amino acids such as lysine and methionine are present in kombucha and are essential for various metabolic processes [101]. Enzymes such as amylase, invertase, and protease are produced during fermentation and can aid in digestion. The diverse bioactive metabolites in kombucha, including organic acids, polyphenols, vitamins, and enzymes, contribute to its potential health benefits and desirable sensory properties [102]. These compounds are produced through the complex interactions between the microbial cultures and the tea substrate during fermentation.

The beneficial microorganisms of kombucha and its antimicrobial properties make it a promising beverage for supporting gut health. As with any dietary supplement, it is important to consume kombucha in moderation and consult with healthcare providers, especially for individuals with underlying health conditions.

## 8. Kombucha, a Potential Health-Promoting Beverage (But Not Probiotic)

Kombucha is rich in live microorganisms (yeasts, AAB, and LAB); it is important to note that it is not a probiotic beverage. Inclusive in vitro “probiotic” studies of kombucha microorganisms have been conducted; however, more research is required. Moreover, several strains belonging to the lactobacilli and *Saccharomyces* spp. were suggested regarding their beneficial properties, and some were evaluated as probiotics. Thus, some of the microbial components of kombucha can be characterized as beneficial properties regarding the regulation of gut health.

The principal challenge is that kombucha SCOBY is a consortium of different microorganisms with variable specificity and proportions. According to regulations of the European Food Safety Authority (EFSA), a probiotic is a specific microorganism identified by appropriate biomolecular methods or by whole genome sequencing and bioinformatic analysis regarding safety [103]. The specific strain needs to be presented in the required minimal concentration and be viable at the time of administration; scientific evidence for benefits needs to be documented by appropriate animal model studies and controlled clinical trials [104].

When fermented food products are investigated for their beneficial properties, several of the previously mentioned criteria are difficult to control. First, precise microbial composition and identity of all microorganisms involved in the preparation of fermented beverages need to confirm the safety of all the strains. The fermented food products are considered safe based on long historical application. Neither can we estimate precise numbers of each of the microbial players part of the microbial consortium, nor can we confirm that the microorganisms are live at the time of consumption of fermented products. Yes, we can follow the beneficial properties of the consumption of the fermented beverage; this may be associated with the “probiotics” properties of the present microorganisms, but most probably with the so-called “postbiotic” properties. According to this concept, the beneficial properties of the consumption of fermented food products are a consequence not directly to the live microorganisms [105] but to their metabolites and parts of the dead cells [106]. While specific clinical trials on the effects of kombucha on gut health are limited, the beverage is known to contain beneficial microorganisms on several occasions incorrectly called probiotics, which can support a healthy gut microbiome. Some studies suggest that the potential probiotics in kombucha may contribute to improved digestion and overall gut health [56].

What is, in actuality, kombucha? Is this a beverage containing probiotics and/or postbiotics? Is it functional food? Is it a beneficial beverage with health-promoting properties? We understand that “functional food” refers to foods designed to provide health benefits beyond basic nutrition. These benefits can be associated with the presence of bioactive compounds, vitamins, or minerals to support well-being [107]. On the other hand, “beneficial food” understand the terms that it is broader and refers to any food that offers positive effects on health when consumed as part of a balanced diet, which naturally supports overall health [107]. However, “probiotic food” is a subset of “functional foods” specifically containing live microorganisms (probiotics) that confer health benefits, particularly for gut health, when consumed in adequate amounts [108]. Moreover, in order to be considered a probiotic, the WHO definition that probiotics are live microorganisms that, when administered in adequate amounts, offer a health benefit to the host must be met. This definition emphasizes that probiotics must be alive, consumed in sufficient quantities, and provide measurable health benefits. But an exact taxonomic identification needs to be clearly determined, the safety profile needs to be confirmed, and appropriate clinical studies need to be performed to enhance claimed health-promoting benefits [109]. Thus, fermented food products can be a source of beneficial microbes, some of them with probiotic properties (potential), but they (fermented foods) cannot be considered probiotics. Or simply, there is no equality between the terms fermented food and probiotic food. Fomented food products are excellent gastronomic products that may bring benefits to consumers associated with their microbial composition, including beneficial microbes, metabolites, and bioactive compounds, which have been consumed for millennia and have been part of traditional medicine. However, more studies still need to be performed in order to unveil all the mechanisms behind the benefits of food products.

## 9. Conclusions

Kombucha has evolved from a traditional homemade fermented tea to a globally consumed functional beverage, largely due to its perceived health benefits and increasing scientific interest. Its production relies on a complex fermentation process involving a SCOBY, which contributes to the formation of organic acids and other metabolites. These components are assumed to play a role in gut microbiota modulation, antioxidant activity, antimicrobial effects, and immune system support. However, despite its long history of consumption and emerging scientific evidence, significant knowledge gaps remain regarding the consistency of its health benefits and the potential risks associated with its intake.

One of the key challenges in assessing the potential benefits of kombucha is the inherent variability in its composition. Factors such as tea selection, sugar concentration, fermentation time, and environmental conditions significantly influence the microbial profile and biochemical composition of the final product. This variability makes it difficult to standardize the functional properties of the beverage, particularly in artisanal preparations. While some commercial products undergo more controlled fermentation and quality assurance processes, homemade versions lack regulation, which can lead to gross inconsistencies in microbial composition and the presence of contaminants. Therefore, establishing standardized protocols for kombucha production is crucial for ensuring both efficacy and safety.

In terms of health benefits, research suggests that kombucha possesses antioxidant properties due to the presence of polyphenols and organic acids, which may help combat oxidative stress and inflammation. Additionally, its antimicrobial activity against pathogenic microorganisms highlights its potential as a natural preservative or therapeutic agent. Some strains of bacteria and yeasts found in kombucha have also been proposed to exert probiotic effects. However, robust clinical trials are still needed to confirm these benefits and establish optimal consumption guidelines.

Safety remains a critical aspect of kombucha consumption. While generally considered safe, excessive intake or improper fermentation can lead to adverse effects such as metabolic acidosis, liver toxicity, or contamination with harmful microorganisms. Regulatory measures and public awareness campaigns on safe kombucha preparation, storage, and consumption practices are necessary to mitigate the risks.

Given the increasing demand for functional beverages, kombucha represents a promising candidate for further development in the food and health industries. Future research should focus on characterizing the specific bioactive compounds responsible for their health benefits, standardizing production techniques, and evaluating their effects through well-designed human clinical trials.

In conclusion, while kombucha has a rich history and intriguing potential as a health-promoting beverage, its widespread consumption should be guided by scientific evidence. Ensuring the safety and efficacy of the beverage requires a combination of standardized production methods, regulatory controls, and further research into its perceived health effects. With continued advances in microbiology, food science, and nutrition, kombucha may consolidate its place not only as a traditional beverage but also as a scientifically approved functional food.

## Figures and Tables

**Table 1:** Diversity of yeast, acetic acid bacteria (AAB), and lactic acid bacteria (LAB) involve in production of kombucha and their benefits for the technological production process and for the consumers.

Microbial Group	Genera	Species	Benefits
Yeast	*Zygosaccharomyces*	*Zygosaccharomyces bisporus*	Tolerant to high sugar and acidic environments, making it well-suited for kombucha fermentation with contribution in carbohydrate metabolism and production of flavor compounds, CO_2_, and alcohol
	*Dekkera*		
	*Starmerella*		
	*Galiella*		
	*Hanseniaspora*		
	*Microidium*		
	*Brettanomyces*	*Brettanomyces bruxellensis*	
	*Candida*	*Candida stellata*	
		*Candida krusei*	
Acetic Acid Bacteria	*Acetobacter*	*Acetobacter aceti*	With the ability to produce acetic acid and bacterial cellulose, AAB contributes to the formation of the characteristic biofilm/cellulosic pellicle named SCOBY. The produced acetic acid reduces pH and contributes to safety. AAB are involved in the production of gluconic acid and the oxidation of glucose to gluconic acid, impacting acidity and flavor profile of kombucha. AAB are responsible for the conversion of ethanol into acetic acid and reducing alcohol levels in final product.
	*Gluconobacter*	*Gluconobacter oxydans*	
	*Swingsia*		
	*Komagataeibacter*	*Komagataeibacter rhaeticus*	
	*Asaia*		
	
	
	
	
Lactic Acid Bacteria	*Lactiplantibacillus*	*Lactiplantibacillus plantarum*	LAB contributes to production of lactic acid, which causes the tangy flavor, reduction in pH, and production of different metabolites with contribution to the flavors and varieties of antimicrobials, including lactic acid, diacetyl, low molecular metabolites, bacteriocins, carbon dioxide, and hydrogen peroxide. Some LAB can have potential health-promoting benefits.
	*Lacticaseibacillus*	*Lacticaseibacillus casei*	

## Data Availability

No new data were created or analyzed in this study. Data sharing is not applicable to this article.
